# Patient‐specific geometrical distortion corrections of MRI images improve dosimetric planning accuracy of vestibular schwannoma treated with gamma knife stereotactic radiosurgery

**DOI:** 10.1002/acm2.14072

**Published:** 2023-06-22

**Authors:** Mojtaba Safari, Ali Fatemi, Younes Afkham, Louis Archambault

**Affiliations:** ^1^ Département de physique de génie physique et d'optique et Centre de recherche sur le cancer Université Laval Québec Québec Canada; ^2^ Service de physique médicale et de radioprotection Centre Intégré de Cancérologie CHU de Québec‐Université Laval et Centre de recherche du CHU de Québec Québec Québec Canada; ^3^ Department of Physics Jackson State University Mississippi USA; ^4^ Merit Health Central Department of Radiation Oncology Gamma Knife Center Mississippi USA; ^5^ Clinical Research Development Unit of Tabriz Valiasr Hospital Tabriz University of Medical Science Tabriz Iran

**Keywords:** deep learning, field mapping, image quality, precision radiation therapy, susceptibility variations

## Abstract

**Purpose:**

To investigate the impact of MRI patient‐specific geometrical distortion (PSD) on the quality of Gamma Knife stereotactic radiosurgery (GK‐SRS) plans of the vestibular schwannoma (VS) tumors.

**Methods and materials:**

Three open access datasets including the MPI‐Leipzig Mind‐Brain‐Body (318 patients), the slow event‐related fMRI designs dataset (62 patients), and the VS dataset (242 patients) were used. We used first two datasets to train a 3D convolution network to predict the distortion map of third dataset that were then used to calculate and correct the PSD. GK‐SRS plans of VS dataset were used to evaluate dose distribution of PSD‐corrected MRI images. GK‐SRS prescription dose of VS cases was 12 Gy. Geometric and dosimetric discrepancies were assessed between the dose distributions and contours before and after the PSD corrections. Geometry indices were center of the contours, Dice coefficient (DC), Hausdorff distance (HD), and dosimetric indices were $D_{\mu }$, $D_{max}$, $D_{min}$, and $D_{95\%}$ doses, target coverage (TC), Paddick's conformity index (PCI), Paddick's gradient index (GI), and homogeneity index (HI).

**Results:**

Geometric distortions of about 1.2 mm were observed at the air‐tissue interfaces at the air canal and nasal cavity borders. Average center of the targets was significantly distorted along the frequency encoding direction after the PSD‐correction. Average DC and HD metrics were 0.90 and 2.13 mm. Average $D_{\mu }$, $D_{95\%,}$ and $D_{min}$ in Gy significantly increased after PSD correction from 16.85 to 17.25, 12.30 to 12.77, and from 8.98 to 9.92. $D_{max}$ did not significantly change after the correction. Average TC and PCI significantly increased from 0.97 to 0.98, and 0.94 to 0.96. Average GI decreased significantly from 2.24 to 2.15 after PSD correction. However, HI did not significantly change after the correction.

**Conclusion:**

The proposed method could predict and correct the PSD that indicates the importance of PSD correction before GK‐SRS plans of the VS patients.

## 1 INTRODUCTION

Magnetic resonance imaging (MRI) plays a vital role in treatment of intracranial tumors using precision radiation therapy like Gamma Knife stereotactic radiosurgery (GK‐SRS).^1^ Precision radiation therapy methods require MRI image quality that is markedly higher than diagnostic radiology, especially because it requires high geometrical accuracy.^2^ For instance, the GK‐SRS treatments require sub‐millimeter MRI geometry distortions to deliver the prescribed steep dose to the target and spare the surrounding healthy tissues.^3^

MRI geometrical distortions, including machine‐specific and patient‐specific, will make the difference between a successful treatment and an excessive dose to healthy structures.^4^ Machine‐specific distortions originate from the field inhomogeneity and gradient non‐linearity. In state‐of‐the‐art MRI scanners, those distortions are negligible, especially for brain imaging with small volume close to the MRI scanner isocenter.^5^, ^6^ On the other hand, patient‐specific geometrical distortions (PSD) are due to the susceptibility changes at the air‐tissue boundaries and water/fat frequency shift resulting from the chemical shift artifact. The magnetic susceptibility changes are not negligible at the air‐tissue interfaces, even when MRI sequences with a modified high bandwidth (BW) are utilized.^7^

The GK‐SRS method using 3D MRI contrast‐enhanced T1 (ceT1) is recommended to plan a treatment of vestibular schwannoma (VS; commonly referred to as acoustic neuromas).^8^, ^9^ However, the magnetic susceptibility changes will geometrically distort MR images of the intracranial tumors close to the air cavities like VS tumors. Little attention has been paid to quantifying the impact of these known geometrical distortions on the dose distribution of VS plans treated with the GK‐SRS plan. In this study, our purpose is twofold: (1) propose a new end‐to‐end distortion correction algorithm and (2) use this algorithm to quantify the impact of PSD on GK‐SRS VS plans.

First, we trained a 3D deep learning network to generate the distortion maps for the VS cases. Then, we manually outlined the target and OAR for the corrected and compared them with the target and OAR of the uncorrected MR images that are available with VS dataset. Finally, we generated GK‐SRS plans using the PSD‐corrected dataset and then copied them to the PSD‐uncorrected VS dataset and evaluated dose distribution after the PSD correction.

## 2 MATERIALS AND METHODS

### 2.1 Datasets

Three open access datasets including the MPI‐Leipzig Mind‐Brain‐Body (318 patients),^10^ the slow event‐related fMRI designs dataset (62 patients),^11^ and the Vestibular Schwannoma‐SEG (VS) dataset (242 patients)^12^ were used. The first two datasets contain MRI images including phase images acquired with different echo times, phase‐difference (Δφ) images, and T1 Magnetization Prepared 2‐ RApid Gradient Echo (MP2RAGE). The VS patient dataset contains 3D ceT1 and high‐resolution T2‐weighted images. Institutional Review Board approval was not applicable to this study since the data came from open‐access datasets.

### 2.2 Methods

#### 2.2.1 MR images PSD correction

**FIGURE 1:**
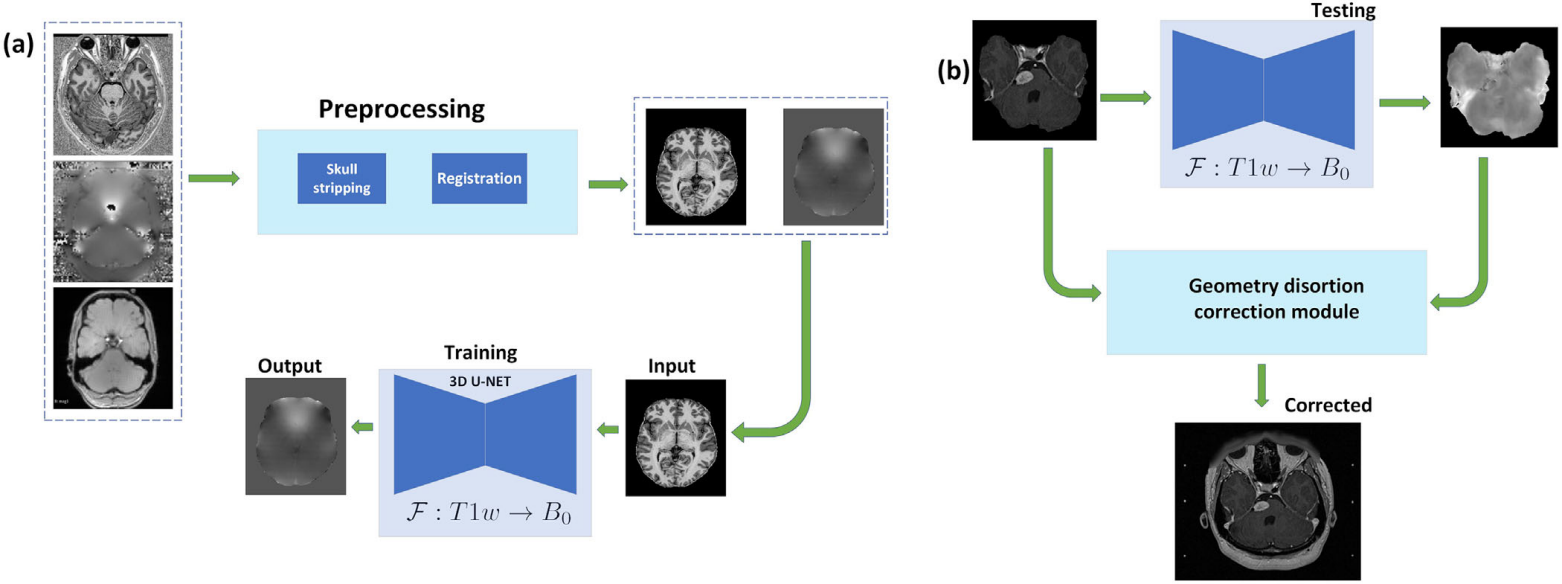
Flowcharts of data preparation (a) and geometry correction (b) are illustrated.

**FIGURE 2:**
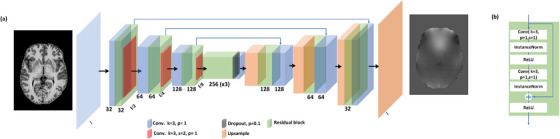
The 3D U‐Net used to estimate the B0 map (a) and the ResNet layer (b) are illustrated. Conv. k = 3, s = 2, p = 1 stands for 3D Convolution layer with 3 × 3 × 3 kernel size, stride 2 × 2 × 2 and padding 1 × 1 × 1 and Conv. k = 3, p = 1 stands for 3D Convolution layer with 3 × 3 × 3 kernel size, stride 1 × 1 × 1 and padding 1 × 1 × 1.

The proposed method is divided into two parts: (1) PSD prediction and correction (see Figure 1) and (2) evaluating geometric and dose distribution changes due to the PSD. After removing the skull from the T1, the magnitude, and the Δφ images using FSL‐BET^1^,^13^ a 3D rigid co‐registration (FSL‐FLIRT^2^ ^13^) with correlation ratio similarity metric was employed to spatially align the magnitude image and T1 images of the same patients. The calculated transform was applied to the Δφ images (see Figure 1a) that is used to estimated distortion map as follows:

(1) $$\Delta B\;=\frac{\Delta \varphi }{\gamma \Delta T_{E}}\;=\frac{\phi \left(T_{E2}\right)-\phi \left(T_{E1}\right)}{\gamma \left(T_{E2}-T_{E1}\right)}\;$$

where $\phi (T_{E2})$ and $\phi (T_{E1})$ are the unwrapped phase images in radians acquired at $T_{E2}$ and $T_{E1}$ given $T_{E2}>T_{E1}$. Finally, the ΔB and T1 were used to train a 3D U‐net^14^ to predict the distortion map from the anatomical MRI images (see Figure 1a).

The 3D U‐Net consisted of three ResNet blocks in the down‐sampling and up‐sampling parts (see Figure 2). Up‐sampling layers were used rather than the transpose convolution layers to preserve the image edge information and avoid the checkerboard effec.^15^ 16 volumetric patches 64×64×64 were randomly extracted from the whole brain volumes to reduce the computation burden and increase the volumetric sample populations.^16^ Thus, the network was trained using 4720 volume samples (corresponding to 295 patients) and validated using 368 volume samples (corresponding to 23 patients) for 150 epochs using Adam optimizer^17^ with learning rate of $2\times 10^{-4}$, β_1_ and β_2_ of 0.9 and 0.999, respectively. The $L_{1}$ regressor was used between the ground truth (reference images) and the network's output (predicted images) since it generates the images with a better resolution. The network was tested using the rest of the two first publicly available databases, the MPI‐Leipzig Mind‐Brain‐Body and the slow event‐related fMRI designs dataset. We implemented the 3D U‐net under the PyTorch 1.10.0^3^ deep learning framework using two NVIDIA GPUs RTX 3090.

To validate the network performance two common quantitative metrics normalized mean square error (NMSE) and the structural similarity (SSIM) were calculated using the validation dataset.^18^, ^19^ The NMSE between the predicted image $\hat{X}\;$and the reference *X* is defined as

(2) $$\mathrm{NMSE}\;=\frac{∥\hat{X}{-X∥}_{2}^{2}}{{∥X∥}_{2}^{2}}\;$$

where ${∥\cdot ∥}_{2}^{2}$ is the squared Euclidean norm, and the subtraction is performed elementwise. The NMSE quantifies the distortion level and lower values indicate lower spatial distortion. The SSIM index with range between 0 and 1, quantifies the structural similarity between the reconstructed image $\hat{X}$ and reference image *X* is defined as follows,

(3) $$\mathrm{SSIM}\;=\frac{\left(2\mu_{X}\mu_{\hat{X}}+c_{1}\right)\left(2\sigma_{X\hat{X}}+c_{2}\right)}{\left(\mu_{X}^{2}+\mu_{\hat{X}}^{2}+c_{1}\right)\left(\sigma_{X}^{2}+\sigma_{\hat{X}}^{2}+c_{2}\right)}\;$$

where$\;\mu_{X}$and $\mu_{\hat{X}}$ are the average voxel values of images *X* and $\hat{X}$, $\sigma_{X}^{2}$ and $\sigma_{\hat{X}}^{2}$ are their variances, $\sigma_{X\hat{X}}^{2}$ is the covariance between them. Two constant variables *c*_1_ and *c*_2_ were used to stabilize the division. This study reported the SSIM using $c_{1}=\;0.01L$ and $c_{2}=\;0.03L$ where *L* was the maximum voxel intensity of reference image *X*.

The second part of the geometry correction involved the distortion map prediction from ceT1 using the network trained in the previous part (see Figure 1b) to correct the PSD. The distortion map of 20 VS cases were predicted using the network and corrected for PSD. Then, they were used to evaluate geometry dose distribution changes after PSD correction. The dosimetric evaluation was done using the GK‐SRS plans generated using PSD‐corrected VS dataset. Calculated per voxel level PSD was applied along the frequency encoding gradient direction as follows,

(4) $$\Delta x\;=\frac{\gamma \Delta B}{2\pi BW}\;$$

where γΔB is given in Equation (1) and BW is in Hz/pixel. In general, geometry distortions stretch or squash a given voxel into a different region that cause the false signal intensity changes. To compensate it, the determinant of the Jacobian matrix J(x,y,z) given in Equation (5) was multiplied by the distorted image^20^, ^21^ assuming the distortions along x, y, and z directions were Δx(x,y,z), Δy(x,y,z), and Δz(x,y,z), respectively .

(5) $$\begin{array}{c} J\;\left(x,y,z\right)=\left(\begin{array}{ccc} 1+\partial \left(\Delta x\right)/\partial x & \partial \left(\Delta x\right)/\partial y & \partial \left(\Delta x\right)/\partial z\\ \partial \left(\Delta y\right)/\partial x & 1+\partial \left(\Delta y\right)/\partial y & \partial \left(\Delta y\right)/\partial z\\ \partial \left(\Delta z\right)/\partial x & \partial \left(\Delta z\right)/\partial y & 1+\partial \left(\Delta z\right)/\partial z \end{array}\right) \end{array}$$

where ∂(Δx)/∂x is a point derivation calculated as $[\Delta x(x_{i+1},\;y_{j}$, $z_{k}$)—$\Delta x(x_{i-1},\;y_{j}$, $z_{k}$)] / [$x_{i+1}$—$x_{i-1}$]. For the PSD correction, J(x,y,z) reduces to a diagonal matrix as the distortion is only along a frequency encoding direction.

#### 2.2.2 GK‐SRS plan

We utilized PSD‐corrected MRI images to generate GK‐SRS plans for all patients. Prior to the procedure, a frame was attached to the patient's head using local anesthesia to numb the scalp. An indicator box was then attached to the head frame, which served as localizers for all patients during GK‐SRS planning using Leksell GammaPlan software V11.1.3 (Elekta, Stockholm, Sweden). The GK‐SRS plans were created by an experienced, board‐certified medical physicist. We imported the MRI images and utilized ball‐bearing markers to define the GK‐SRS coordinates. Next, we simulated the head contour to generate the entire head contour automatically. Finally, we drew a target volume based on the tumor visualized on ceT1 images.

The highly conformal GK‐SRS plans were achieved by outlining the target with multiple isocenters of the same or different diameters to cover the entire tumor volume with the 50% isodose line with a 12 Gy prescription dose.

To quantify the impact of the PSD on the targets and dose distribution of the GK‐SRS plans, geometric and dosimetric metrics were reported. Geometric indices were volume of the target, center of the target, sørensen–Dice coefficient (DC), and the Hausdorff distance (HD). The dosimetric indeces were mean dose ($D_{\mu }$), maximum dose (*D*_max_), minimum dose (*D*_min_), $D_{95\%}$, target coverage (TC, higher is better), Paddick's conformity index (PCI, higher is better),^22^ Paddick's gradient index (GI, lower is better),^23^ and homogeneity index (HI).^24^

This study used the Wilcoxon signed‐rank test to evaluate the null hypothesis that quantitative metrics of PSD‐corrected and PSD‐uncorrected were similar (*P* ≥0.05). *P*‐value < 0.05 was considered statistically significant.

## 3 RESULTS

### 3.1 Network evaluation

**FIGURE 3:**
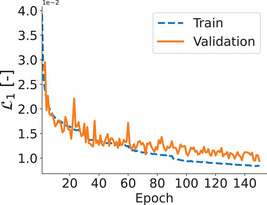
Training and validation losses are illustrated where small gap between training loss and validation loss guarantees that the model did not overfit.

**FIGURE 4:**
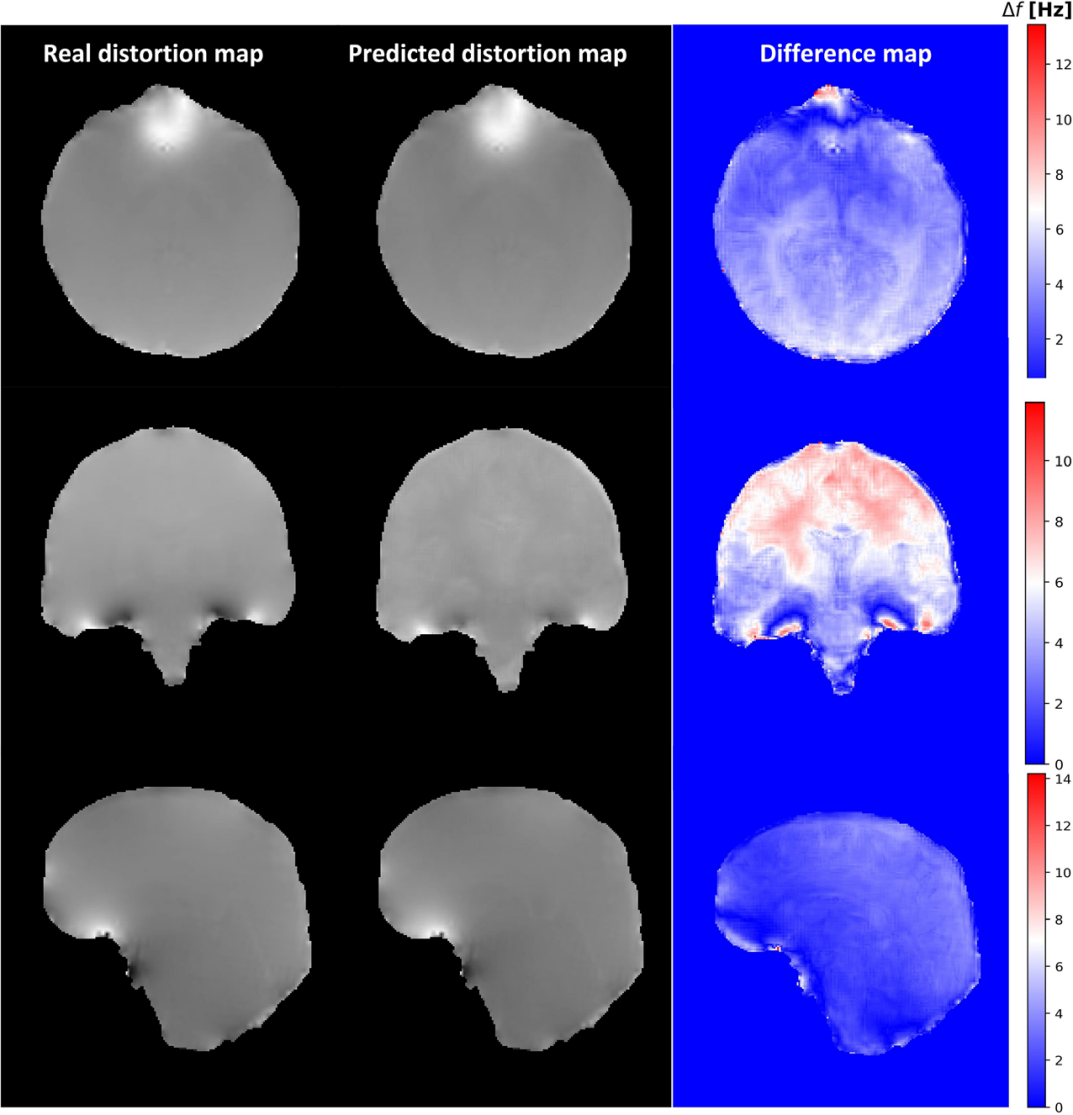
Comparison between real distortion map and predicted distortion map for a representative patient is illustrated for axial, sagittal, and coronal views.

The $L_{1}$ train and validation losses are shown in Figure 3 where small gap between training and validation loss guarantee that our network did not overfit the data. The comparison between real distortion map and predicted distortion map and the difference map for axial, coronal, and sagittal views using our proposed 3D U‐Net are shown for one representative patient in Figure 4.

The NMSE and the SSIM indices were 6.37%±0.59% and 0.97 ± 0.01, respectively. Small NMSE expresses that the reconstructed images contain small level of spatial distortion. In addition, large SSIM index indicates a high structural resemblance between predicted and reference images.

### 3.2 Geometric evaluation

**FIGURE 5:**
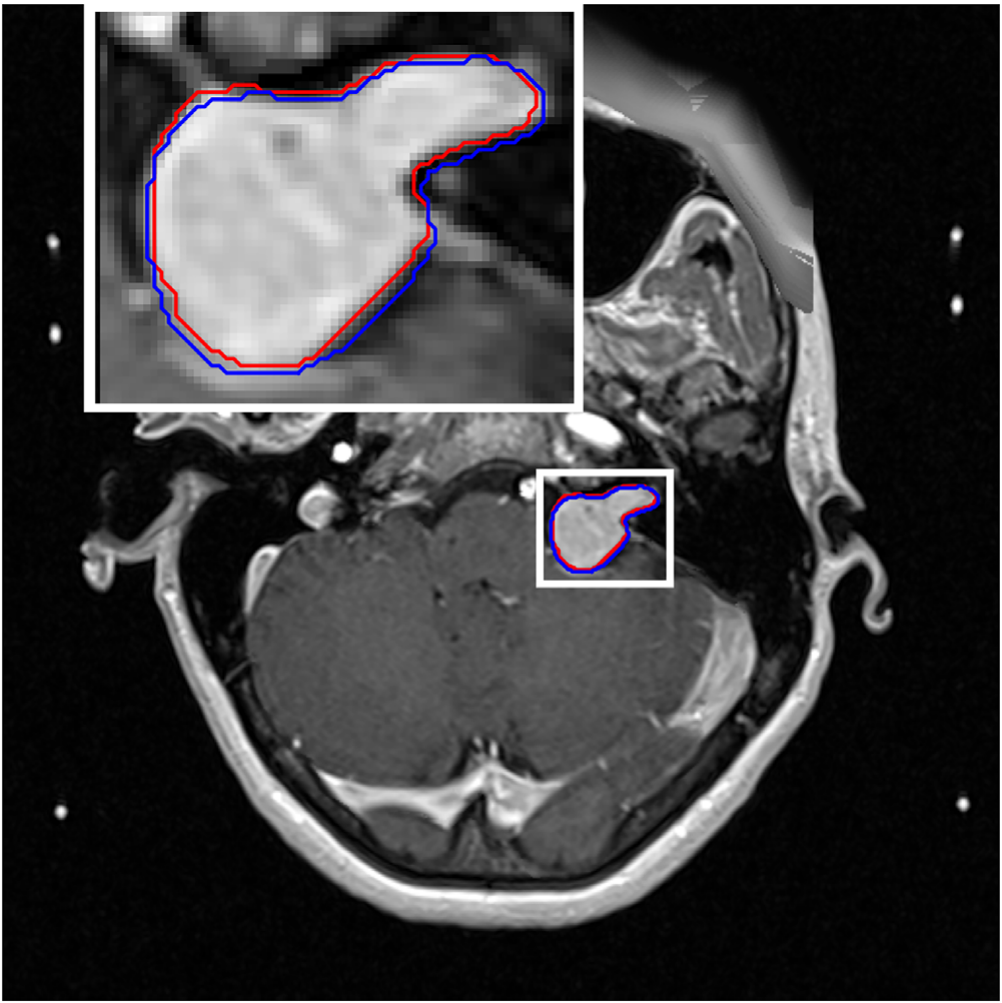
PSD‐corrected ceT1 with the contours manually outlined on the PSD‐corrected (red) and non‐corrected (blue) images with a maximum shift of 0.9 mm are illustrated. The top left is the zoomed image outlined with a white box around the tumor.

To determine the impact of PSD on the GK‐SRS plans of the VS patients, we employed the predicted PSD to correct the ceT1 images. We found the center of the manual contours significantly distorted along the frequency encoding direction (P=0.0005) but not along the phase encoding directions (see Figure 5). In addition, the target volume was significantly increased from 2.72 ± 0.71 cc to 2.95 ± 0.74 cc (P=0.0002) after correcting the PSD.

**FIGURE 6:**
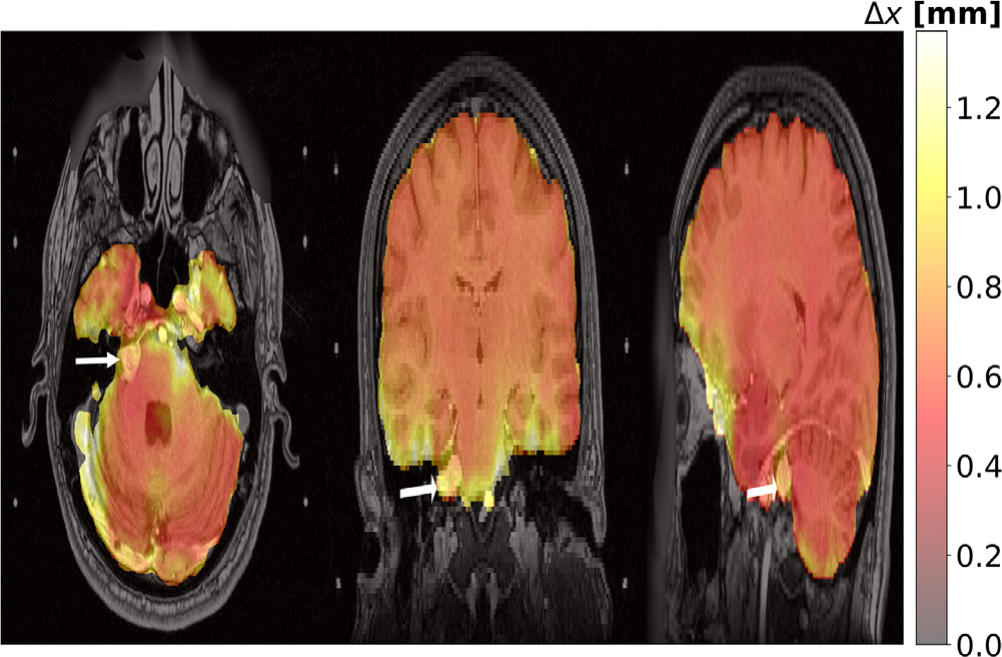
The PSD non‐corrected ceT1 data overlaid by the distortion map in mm is illustrated for three views. The white arrows indicate the VS tumor.

Similar to the susceptibility artifact reported at the air‐tissue interfaces,^7^, ^25^, ^26^ the peripheral regions of the brains (air‐tissue interfaces) had marked distortions of around 1.2 mm (Figure 6), where the VS tumors are located. To determine the similarity between the contours before and after PSD corrections are reported: sørensen–Dice coefficient (DC) and the Hausdorff distance (HD) metrics. The DC similarity score between the contours before and after PSD correction was 0.90 ± 0.012 (95% CI = 0.87 − 0.93), indicating a moderate similarity. The HD metric was up to 2.13 ± 0.22 mm (95% CI = 1.65 − 2.62 ). The moderate DC and large HD values show the importance of the PSD.

### 3.3 Dosimetric evaluation

**FIGURE 7:**
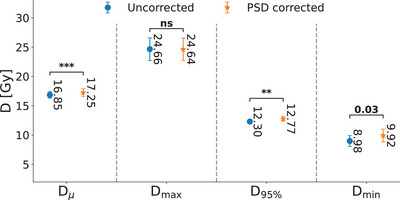
Dosimetric indices including $D_{\mu }$, $D_{95\%}$,$\;D_{max}$, and $D_{min}$ are illustrated for the datasets before and after the PSD correction. The p‐values were estimated using the Wilcoxon signed‐rank test.

After correcting the PSD, $D_{\mu }$, $D_{95\%}$, and *D*_min_ significantly increased from 16.85 ± 0.56  to 17.25 ± 0.68 Gy (P<0.001), 12.30 ± 0.30  to 12.77 ± 0.41 Gy (P<0.01), and 8.98 ± 0.94  to9.92 ± 1.11 Gy (P=0.03), respectively. The reduction in minimum dose of PSD uncorrected data is due to the targets’ translation along frequency encoding direction after PSD‐correction, thus, the regions with lower dose at peripheral regions were inside the targets after PSD‐uncorrected data. In addition, the increase of $D_{95\%}$ represents a better tumor coverage. Also, the *D*_max_ did not significantly change (*P* = 0.3) after correcting the geometry distortion as the hot‐spots are formed within the target and will not be affected by PSD distortion at the air‐tissue interfaces. The dosimetric indices are presented in Figure 7.

**FIGURE 8:**
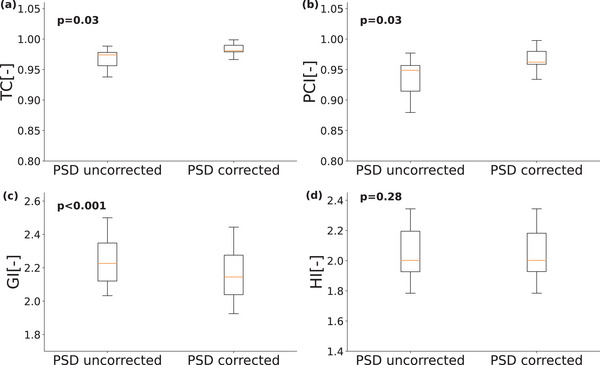
Dosimetric indices are illustrated including (a) target coverage, (b) Paddick's conformity index, (c) Paddick's gradient index, and (d) homogeneity index.

Finally, we observed significant changes in the dosimetric indices calculated from dose distributions including TC, PCI, and GI. The TC significantly increase from 0.97 ± 0.02 to 0.98 ± 0.03 (P=0.03) after the PSD correction (Figure 8a). A similar trend was observed for the PCI where it significantly increased from 0.94 ± 0.03 to 0.96 ± 0.03 (P=0.03) (Figure 8b). The significant increase of both metrics represents a better tumor coverage. Conversely, GI index significantly reduced from 2.24 ± 0.15 to 2.15 ± 0.15 (P<0.001), however, HI did not change significantly (P=0.3), respectively, after the PSD correction (Figure 8c,d). Those reductions implied a less steep dose distribution with a similar homogeneous dose distribution.

## 4 DISCUSSION

This is the first study to investigate the impact of PSD on the quality of MRI‐based GK‐SRS plans. Our findings emphasize the necessity of PSD correction for VS patients and generally for GK cases with a target close to the air cavities. Furthermore, we showed that the geometric and dosimetric indices of the GK‐SRS plans were significantly changed after the PSD corrections.

Similar to a phantom study^27^ and an in vivo study,^7^ we found that magnetic susceptibility changes cause a geometry distortion of around 1.2 mm in the peripheral regions at the tissue‐air interfaces (Figure 6). Furthermore, we found that the PSD significantly reduces the tumor volumes and distorts the contours along the frequency encoding direction. In addition, moderate DC and large HD values indicate a large geometry difference due to the PSD that make the correction crucial for VS patients because the positional errors are larger than the tolerable ±1 mm for GK‐SRS plans.^28^, ^29^

Moreover, we reported dosimetric indices including CI, PCI, GI, and HI extracted from dose distributions and the dose values including $D_{\mu }$, $D_{max}$, $D_{min}$, and $D_{95\%}$. We found statistically significant reduction in the quality of dosimetric values shown in Figures 7 and 8. The dose values indicate a worse tumor coverage ($D_{95\%}$) and lower dose delivered to the cold‐spots ($D_{min}$) as the targets were translated due to the PSD far from of PSD‐uncorrected target volumes. Those findings follow the dosimetric indices as the reductions of the CI and PCI indicate worse tumor coverage, and the increase of the GI result in less steep dose distributions in the tumor. Thus, PSD corrections would be required otherwise the dose distribution quality could deteriorate. We believe the most predominant effect of PSD is the target deformation and specifically its geometric distortion at air‐tissue interfaces which are dosimetrically significant for SRS treatments. The PSD is considered a deformable distortion that requires a computationally expensive multi‐modal deformable co‐registration. Compared with the current clinical settings, the significances of our method are three‐fold; (a) it is computationally cheap; (b) it is free from delivering radiation dose to the patient; and (c) it is only required to obtain fast images with an acquisition time of under one minute.

This study has some limitations that should be discussed. We did not account for the geometric distortion caused by the administration of Gd contrast in a B0 magnetic field. This type of distortion can reach a maximum value of 0.5 mm,^30^ and correcting for it would require acquiring a second set of identical ceT1 images but with a reverse gradient, effectively doubling the imaging acquisition time.

## 5 CONCLUSION

The proposed method could predict and correct the PSD. It indicates the importance of PSD correction before GK‐SRS plans of the VS patients. The proposed method is fast, computationally cheap, easy to implement, and applicable to any MRI dataset. In addition, it will improve the standard of care of many radiation therapy clinics that do not have the expertise to run specific MRI pulse sequences and post‐processing for MRI patient‐specific geometrical distortion.

## AUTHOR CONTRIBUTIONS

The authors confirm their contribution to the paper as follows. Study conception and design: Ali Fatemi. Collection and assembly of data: Mojtaba Safari and Younes Afkham. Analysis and interpretation of results: Mojtaba Safari, Ali Fatemi, and Louis Archambault. Draft manuscript preparation: Mojtaba Safari, Ali Fatemi, and Louis Archambault. Final approval of the version to be published: Louis Archambault, Ali Fatemi, Younes Afkham, and Mojtaba Safari. All authors reviewed the results and approved the final version of the manuscript.

## CONFLICT OF INTEREST STATEMENT

The authors declare that the research was conducted in the absence of any commercial or financial relationships that could be construed as a potential conflict of interest.

## Data Availability

All data are publicly available at:
TCIA dataset: https://wiki.cancerimagingarchive.net/pages/viewpage.action?pageId = 70229053 OpenNeuro dataset: https://openneuro.org/datasets/ds000221/versions/1.0.0 and https://openneuro.org/datasets/ds004349/versions/1.0.0

## Footnotes

1
https://fsl.fmrib.ox.ac.uk/fsl/fslwiki/BET

2
https://fsl.fmrib.ox.ac.uk/fsl/fslwiki/FLIRT

3
https://pytorch.org/
